# Physical activity and body dissatisfaction among female university students: a three-wave study of psychological flexibility and resilience

**DOI:** 10.3389/fpsyt.2026.1960191

**Published:** 2026-10-14

**Authors:** Youshan Zhang, Zhigang Liu, Chang Hu, Laibing Lu

**Affiliations:** 1 Department of Tactics, Fujian Police College, Fuzhou, China; 2 Physical Education College, Hunan First Normal University, Changsha, China; 3 Physical Education College, Jiangxi Normal University, Nanchang, China; 4 Department of Sports Training, Henan Sport University, Zhengzhou, China

**Keywords:** body dissatisfaction, physical activity, psychological flexibility, psychological resilience, time-lagged indirect association

## Abstract

**Objective:**

Body dissatisfaction (BD) is a common psychological concern among female university students. Although physical activity (PA) has been associated with more favourable body-related evaluations, the psychological factors that may account for this association remain unclear. This study examined the independent and theoretically ordered indirect associations of psychological flexibility (PF) and psychological resilience (PR) in the prospective association between PA and BD.

**Methods:**

A three-wave prospective study was conducted among 1,086 first- and second-year female university students from three universities in China. Assessments were administered at 6-month intervals over 12 months. The hypothesised statistical model included T1 PA, T2 PF, and T3 PR and BD. PF was operationalized as lower experiential avoidance, with lower experiential avoidance representing greater PF. Only-child status, BMI category, and self-rated health were included as covariates. Indirect associations were estimated using PROCESS Model 6 with 5,000 bootstrap resamples.

**Results:**

T1 PA was positively associated with T2 PF (*β* = 0.419, *p* <.001) and T3 PR (*β* = 0.239, *p* <.001), while T2 PF was positively associated with T3 PR (*β* = 0.434, *p* <.001). T1 PA, T2 PF, and T3 PR were each negatively associated with T3 BD (*β* = −0.127, −0.225, and −0.355, respectively; all *p* <.001). The total indirect association was significant (*β* = −0.243, 95% bootstrap CI [−0.280, −0.206]). Significant specific indirect associations were observed through PF (*β* = −0.094, 95% bootstrap CI [−0.121, −0.069]), through PR (*β* = −0.085, 95% bootstrap CI [−0.109, −0.061]), and through the theoretically ordered PF–PR sequence (*β* = −0.064, 95% bootstrap CI [−0.081, −0.050]).

**Conclusions:**

Higher PA was prospectively associated with lower subsequent BD, with significant theoretically ordered indirect associations involving lower experiential avoidance, operationalized as greater PF, and greater PR. These findings highlight the potential relevance of adaptive psychological resources in understanding the prospective association between PA and BD among female university students.

## 1 Introduction

Body dissatisfaction (BD), characterised by a persistent negative evaluation of one’s body size, shape, weight, or overall appearance, is an important psychological health concern during the transition to young adulthood (1, 2). Longitudinal population evidence shows that dissatisfaction commonly persists or increases from adolescence into young adulthood (3). Female university students may be particularly vulnerable because university entry coincides with changes in social relationships, lifestyle patterns, and self-identity, while increasing exposure to appearance comparison and sociocultural ideals surrounding thinness and fitness (4). Experimental studies show that brief exposure to appearance-focused social media can increase appearance comparison and reduce body satisfaction in college women (5, 6), and a systematic review similarly linked appearance-focused social-networking activities with body-image concerns and disordered eating (7). BD is therefore not merely an aesthetic concern: prospective evidence identifies it as a shared risk factor for later depressive symptoms, disordered eating, and weight-related difficulties (8–10). Identifying modifiable factors associated with lower subsequent BD is consequently important for protecting female students’ psychological and behavioural health.

Physical activity (PA) represents a potentially modifiable lifestyle factor associated with both physical and psychological functioning (11–14). Exercise-intervention meta-analyses have generally identified small improvements in body image, and a recent female-specific synthesis reported an overall beneficial effect on body esteem (15–17). A broad scoping review likewise concluded that PA and sport participation were more often related to less negative and more positive body image, while also emphasising the scarcity of studies capable of establishing directionality (18). The association is not uniformly protective. Among college women, appearance-based exercise motivation weakens the positive association between exercise frequency and body image, and daily-life benefits are smaller when exercise increases appearance awareness. Longitudinal evidence further suggests that low body satisfaction can precede increased activity in weight-concerned college women, and that BD and excessive exercise can reinforce one another (19, 20). These findings indicate that the psychological meaning and regulatory processes accompanying PA may be as important as the amount of activity itself. Nevertheless, prospective mechanisms linking PA with later BD remain insufficiently understood.

Existing research has mainly explained body-image outcomes in relation to sociocultural pressure, appearance comparison, thin-ideal internalisation, self-esteem, and exercise motivation. Although valuable, these perspectives provide limited insight into how adaptive psychological capacities may translate PA participation into more favourable body evaluations over time. Psychological flexibility (PF) refers to the capacity to remain open to difficult internal experiences, maintain contact with the present moment, and pursue behaviour aligned with personally meaningful values (21–23). In the context of body image, greater PF may allow students to experience appearance-related thoughts or negative social comparisons without becoming rigidly fused with them or resorting to avoidance. An intervention study in Chinese university students found that cognitive defusion and PF predicted negative body image and its improvement (24). At the domain-specific level, body-image flexibility is consistently associated with less negative body image, eating pathology, and psychological distress (24, 25), including in recent longitudinal evidence from Chinese adults (26). PF may therefore represent a proximal self-regulatory process linking PA with body-related cognition and emotion.

Psychological resilience (PR) reflects a dynamic capacity to adapt and recover when confronted with stress or adversity (27). Female university students frequently encounter appearance criticism, social comparison, and perceived discrepancies between their actual and ideal bodies. Students with stronger PR may be better able to treat these experiences as manageable rather than allowing them to consolidate into persistent negative body evaluations. PA can provide repeated opportunities to manage discomfort, overcome challenges, and develop mastery. Recent two-wave and experimental studies in Chinese college students link exercise with greater subsequent PR and suggest that PR may statistically account for part of the association between exercise and mental-health outcomes (28). Direct body-image evidence is emerging: a three-wave random-intercept cross-lagged study found that PR mediated the association between self-esteem and later body image among Chinese college students (29), while the body-image resilience model identifies coping resources and lower sociocultural pressure as protective for first-year college women (30). PF may precede PR because open, values-guided responses can supply the regulatory basis for adaptive recovery. However, although ACT interventions reliably improve PF in undergraduates, direct longitudinal evidence that PF develops into PR remains limited (31, 32).

Accordingly, the present study employed a three-wave longitudinal design to examine the prospective association between PA and BD among female university students and to investigate the separate and theoretically ordered indirect associations involving PF and PR. The proposed statistical model assumes that greater PA is prospectively associated with higher subsequent PF, that higher PF is associated with greater PR, and that greater PR is associated with lower BD. By assessing the study variables across three measurement waves, this research extends prior cross-sectional evidence by examining theoretically ordered, time-lagged associations among PA, PF, PR, and BD. The findings may extend current understanding of the interplay among PA, adaptive psychological functioning, and BD, while informing future research and the development of university-based approaches that consider PA alongside psychological flexibility and resilience.

## 2 Theoretical foundation and research hypotheses

### 2.1 Relationship between PA and BD

PA refers to any bodily movement produced by skeletal muscles that results in energy expenditure and includes exercise, active transportation, recreational activity, and other forms of daily movement (33). From an embodiment perspective, body evaluations are shaped not only by appearance information but also by direct bodily experience. Regular PA gives female university students opportunities to experience strength, endurance, coordination, and physical competence. These experiences may help them recognise the functional value of their bodies and shift attention from how their bodies appear to what they can accomplish, a proposed explanation consistent with evidence that PA and sport are more often associated with favourable body-image outcomes.

Female university students are frequently exposed to appearance ideals, social comparison, and appearance-based evaluation, which may increase perceived actual–ideal discrepancies and contribute to BD (34). Objectification theory proposes that repeated evaluation of the body from an observer’s perspective encourages body surveillance and persistent appearance monitoring (35). PA may counter this process when it increases bodily engagement, perceived competence, and awareness of physical functioning. Mastering physical tasks and coping with challenges may broaden the bases of self-evaluation beyond appearance. PA can also provide emotional release, social interaction, and stress regulation, although these benefits depend on how the activity is experienced and interpreted.

The association between PA and BD may therefore depend on the meaning attached to participation. PA undertaken primarily for weight control or appearance modification can reinforce body monitoring, self-objectification, and eating concerns, whereas activity motivated by health, enjoyment, competence, and personal growth is more often associated with favourable body-related outcomes (36, 37). Daily-life evidence further shows that appearance-motivated exercise produces smaller gains in state body satisfaction (38). Despite this complexity, exercise-intervention meta-analyses generally identify a small average improvement in body image (17). A longitudinal examination is needed to test whether higher PA is associated with lower later BD in female university students. Accordingly, the first hypothesis is proposed as follows:

*H1. Higher PA will be prospectively associated with lower subsequent BD among female university students.*

### 2.2 The mediating role of PF

PF refers to an individual’s capacity to remain open to difficult thoughts, emotions, and bodily sensations, maintain awareness of present-moment experience, and persist in or adjust behaviour in accordance with personally meaningful values (39). Rather than attempting to eliminate unpleasant internal experiences, individuals with higher PF can allow these experiences without letting them rigidly determine self-evaluation or behavioural choice. In the body-image context, this capacity may reduce the likelihood that temporary dissatisfaction develops into a persistent negative evaluation of the body (40).

PA may be a behavioural context in which flexible responding is practised. Participation often requires tolerating physical discomfort, regulating frustration, attending to ongoing bodily experience, and continuing toward meaningful goals (41, 42). Repeated effort and adjustment may strengthen willingness to experience discomfort without avoidance and may support values-guided action, processes central to the PF model (43). When PA is undertaken for health, enjoyment, or personal growth rather than appearance correction, it may also shift attention away from rigid appearance standards (44). Therefore, higher PA may be associated with greater subsequent PF, although direct longitudinal evidence for this link is still limited.

PF may, in turn, be negatively associated with BD. Individuals with lower flexibility may respond to idealised appearance standards and self-critical thoughts through cognitive fusion, experiential avoidance, and rigid attempts to control appearance. By contrast, greater flexibility may help students recognise body-critical thoughts as transient mental events and continue valued academic, social, and health-related activities despite temporary discomfort. Body-image-specific flexibility is inversely associated with dissatisfaction and disordered eating across measurement, college, and meta-analytic studies (25, 45, 46), and it can buffer eating risk in women (47). Recent cross-lagged evidence further suggests bidirectional links between flexibility and psychological/eating-related difficulties in Chinese adults (26).

PF may therefore constitute a proximal mechanism linking PA with BD. Greater PA may provide opportunities to practice present-moment awareness, acceptance, and behavioural adjustment, while greater PF may weaken the influence of appearance pressure on self-evaluation. Because the PA–body-image literature remains dominated by cross-sectional designs and has rarely tested mechanism-oriented longitudinal models, the temporal role of PF requires direct examination. Based on the above reasoning, the following hypotheses are proposed:

*H2. Higher PA will be prospectively associated with higher subsequent PF among female university students.*
*H3. PF will show a significant indirect association between PA and BD, with higher PA associated with higher PF and higher PF associated with lower BD.*

### 2.3 The mediating role of PR

PR refers to the capacity to adapt effectively, recover, and maintain functioning when confronted with stress or significant challenges (27). It is better understood as a dynamic adaptive process than as the absence of distress. In the body-image context, appearance comparison, idealised standards, and negative comments can produce substantial stress (48, 49). Greater PR may support emotional regulation, balanced appraisal, and recovery, reducing the likelihood that temporary appearance concerns consolidate into enduring BD (50).

PA may provide manageable challenges through which PR develops. Activity requires persistence, behavioural adjustment, and coping with discomfort or setbacks; successful engagement can strengthen mastery, confidence, and adaptive coping. Organised PA may additionally provide social support and belonging (51–53). These propositions are consistent with resilience theory, in which adaptive capacity develops through engagement with challenge (54), and with recent longitudinal and experimental evidence linking exercise with PR among Chinese college students (55).

PR may, in turn, be negatively associated with BD. Students with lower PR may interpret appearance criticism or unfavourable comparison as stable and personally defining, whereas students with greater PR may recover more rapidly and draw on alternative sources of self-worth. The body-image resilience model identifies active coping, support, and lower sociocultural pressure as contributors to more positive body image in first-year college women (30). More recently, three-wave evidence from Chinese college students showed that PR mediated the association between self-esteem and later body image, and the PRISM framework emphasised that resilience should be paired with interpersonal and systemic responses to weight stigma (56).

Accordingly, PR may be one mechanism connecting PA with BD: higher PA may supply mastery and coping experiences associated with greater PR, while PR may help students withstand appearance-related stress. Existing PA–PR studies have largely focused on depression, anxiety, or general mental health rather than body image, and direct body-image work has examined other antecedents such as self-esteem. The proposed PA–PR–BD pathway, therefore, integrates adjacent literatures and requires longitudinal testing.

*H4. Higher PA will be prospectively associated with higher subsequent PR among female university students.*
*H5. PR will show a significant indirect association between PA and BD, with higher PA associated with higher PR and higher PR associated with lower BD.*

### 2.4 The sequential mediating roles of PF and PR

PF represents an adaptive self-regulatory capacity that enables openness to difficult internal experiences and values-consistent action (57, 58). This capacity may provide a foundation for PR because students who are less entangled with negative thoughts and more willing to experience discomfort can preserve behavioural options under stress. In the body-image context, flexible responding may help students avoid treating criticism, comparison, or self-critical thoughts as definitive judgments of personal worth (59, 60).

PF may promote PR by reducing experiential avoidance and rigid responding. When individuals can allow unpleasant thoughts and emotions without being controlled by them, they may appraise stressors more flexibly, select appropriate coping strategies, and continue to pursue meaningful goals (61). Repeated use of these regulatory processes could accumulate into a broader capacity for recovery, consistent with developmental accounts of resilience as adaptation supported by ordinary regulatory resources (54). This PF-to-PR ordering is theoretically plausible, but it has received little direct longitudinal testing.

PA may initiate this adaptive process by providing repeated experiences of effort, discomfort, challenge, and achievement. During activity, students remain engaged with bodily sensations, regulate frustration, and adjust behaviour toward valued goals. These experiences may support PF; repeated flexible responses may then broaden into PR, which may protect against persistent BD (62). Evidence that ACT interventions improve PF in university students supports the modifiability of the first psychological process, while longitudinal work supports PR as a pathway to later body image from other personal resources (29). No prior evidence directly establishes the complete PA–PF–PR–BD sequence.

Previous research has separately connected PA with body image and PR, and PF with body-related outcomes, but the internal ordering of PF and PR has received limited attention. Treating them as parallel mediators may overlook the possibility that PF is a proximal regulatory process through which repeated activity experiences are translated into broader resilience. Conversely, PR could precede PF, or both could reflect an unmeasured resource. Testing the proposed sequence, therefore, offers a theoretically informative but necessarily provisional extension of the literature. Based on the above reasoning, the following hypothesis is proposed:

*H6. PF and PR will show a significant sequential indirect association between PA and BD, with higher PA associated with higher PF, higher PF associated with higher PR, and higher PR associated with lower BD. Overall*, **Figure 1** *presents the theoretical model of the present study.*

**Figure 1:**
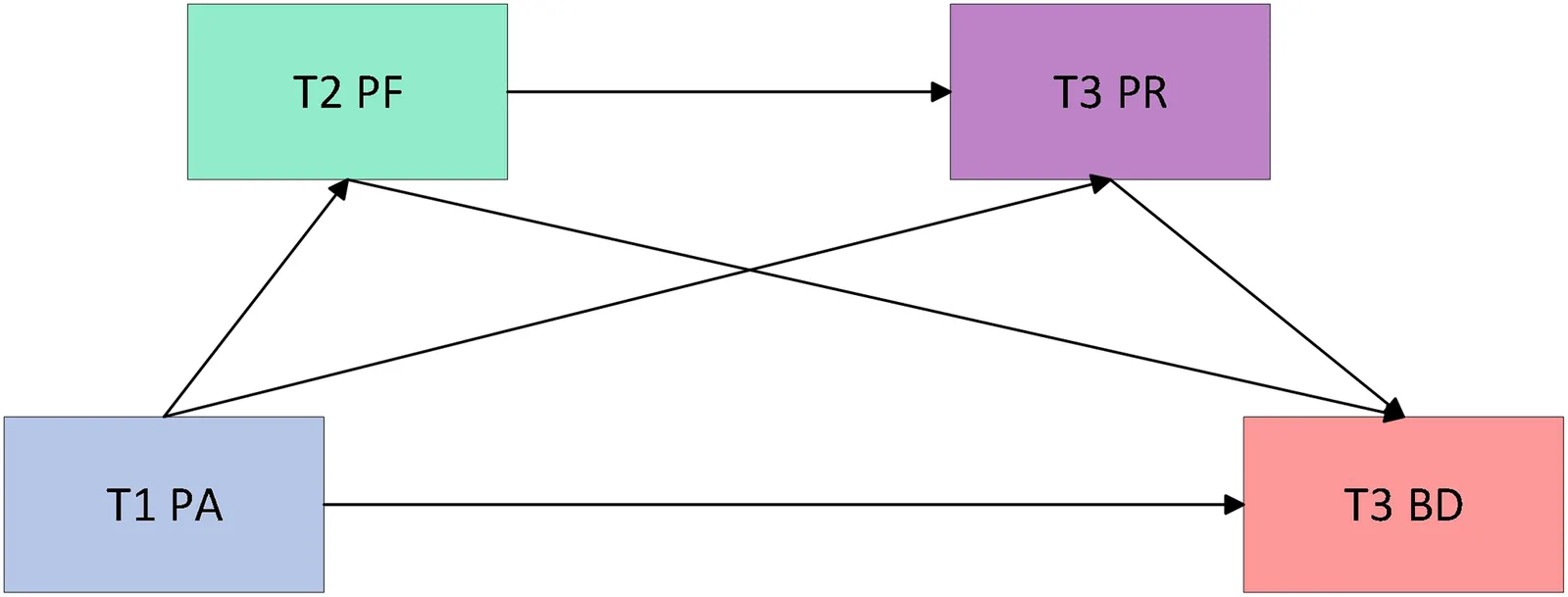
Proposed sequential mediation model PA, physical activity; PF, psychological flexibility; PR, psychological resilience; BD, body dissatisfaction. Arrows depict the hypothesised direct and indirect pathways.

## 3 Methods

### 3.1 Study design

A three-wave prospective longitudinal study was conducted among female university students from three universities in Jiangxi and Henan Provinces, China. Data were collected at 6-month intervals, with the first wave conducted in April 2025 (T1), the second in October 2025 (T2), and the third in April 2026 (T3). PA, PF, PR, and BD were assessed using the same instruments and standardised procedures at all three waves. The study examined the time-lagged association between PA and BD and whether PF and PR statistically accounted for this association, independently and sequentially. Reporting followed the Strengthening the Reporting of Observational Studies in Epidemiology (STROBE) guidance for cohort studies (63).

### 3.2 Participants

Convenience sampling was used to recruit full-time first- and second-year female undergraduate students from three universities located in Jiangxi and Henan Provinces, China. Before participant recruitment, the research team contacted the relevant university administrators and course instructors to explain the purpose and procedures of the study and obtain permission to conduct the survey.

The inclusion criteria were: (1) being a registered full-time female undergraduate student in the first or second academic year; (2) being able to understand and independently complete the Chinese questionnaires; (3) being expected to remain enrolled at the participating university throughout the follow-up period; and (4) understanding the study information and voluntarily agreeing to participate. Students were excluded if they: (1) were on academic leave or had suspended or withdrawn from their studies during the data-collection period; (2) submitted questionnaires that failed the predefined quality-control criteria; or (3) provided identification information that could not be reliably matched across the three measurement waves.

At T1 in April 2025, 1462 participants provided valid baseline data. Among them, 1289 completed the T2 assessment in October 2025, corresponding to a retention rate of 88.17% and an attrition rate of 11.83% from T1 to T2. At T3 in April 2026, 1,127 participants completed the survey, corresponding to a retention rate of 87.43% and an attrition rate of 12.57% from T2 to T3. Relative to the initial T1 sample, the T3 retention rate was 77.09%. After data-quality screening and longitudinal matching, 1086 participants had complete and successfully matched data across all three waves and were included in the final longitudinal analyses, representing 74.28% of the baseline sample. The participant recruitment, attrition, and inclusion process is presented in **Figure 2**.

**Figure 2:**
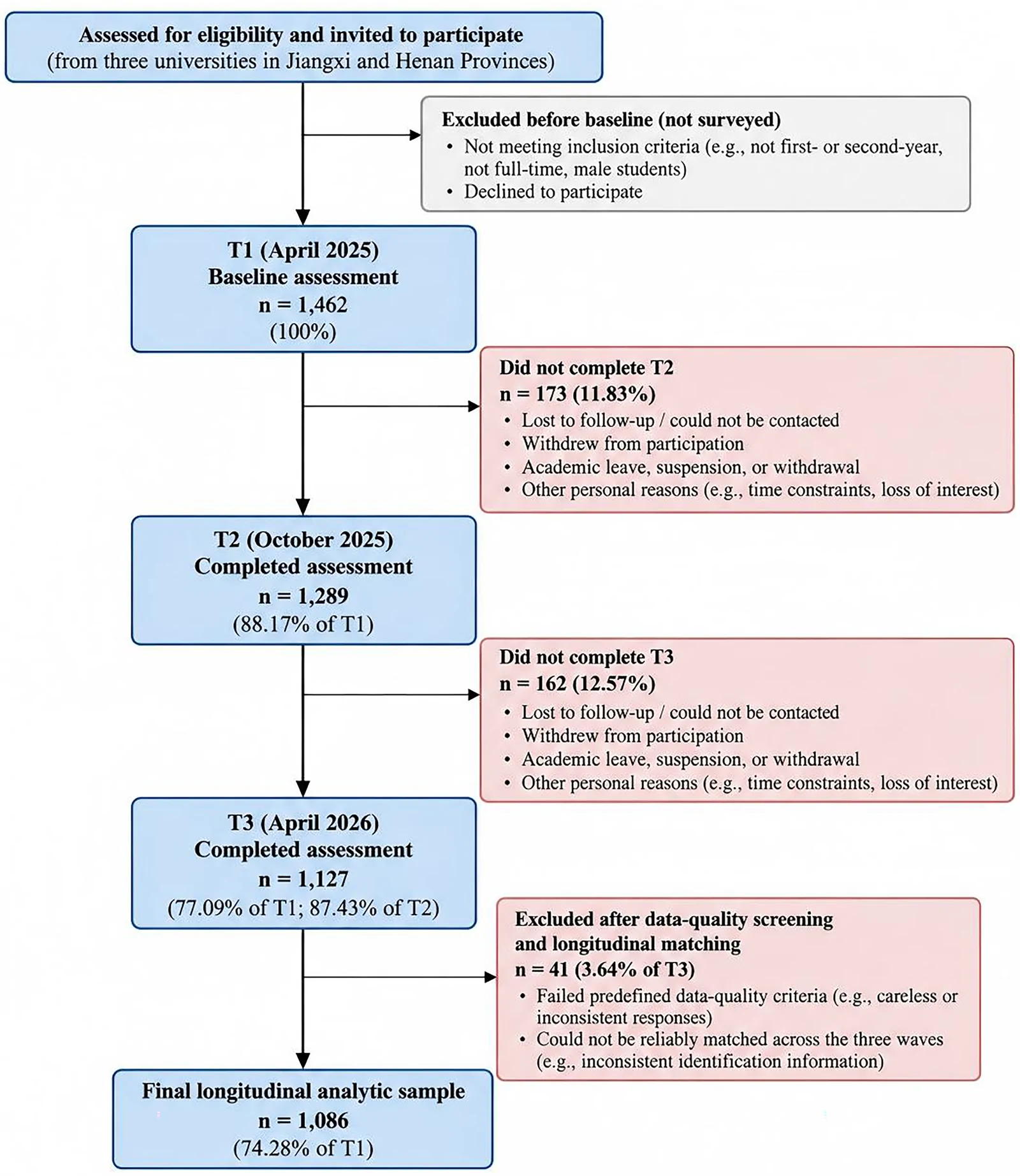
Participant flow across the three-wave longitudinal study.

To examine whether participant attrition was systematic, baseline characteristics were compared between participants who completed all three waves and those who did not provide complete longitudinal data. Independent-samples t-tests were used for continuous variables, and chi-square tests were used for categorical variables. The comparisons included age, academic year, major, place of residence, only-child status, BMI category, perceived family economic status, self-rated health, participation in university sports clubs, monthly living expenses, and baseline PA, PF, PR, and BD scores. No statistically significant differences were observed between retained and non-retained participants for any of the baseline variables examined (all *p* > 0.05), suggesting that there was no evidence of systematic or selective attrition.

### 3.3 Data collection and survey instruments

#### 3.3.1 Participant demographic information questionnaire

The sociodemographic questionnaire was developed following discussions among the research team. It collected information on age, academic year, major, place of residence, only-child status, BMI category, perceived family economic status, self-rated health, participation in university sports clubs, and monthly living expenses. Sex was not included as an analytic variable because all participants were female. Based on theoretical relevance and previous evidence, only-child status, BMI category, and self-rated health were selected *a priori* as covariates because of their potential associations with PA, psychological functioning, and body-related evaluations (13, 64, 65).

#### 3.3.2 Physical activity

PA was assessed using the Physical Activity Rating Scale-3 (PARS-3) (66). The scale comprises three components assessing exercise intensity, duration per session, and frequency, each rated on a 5-point scale. The overall PA score was calculated using the formula: exercise intensity × (exercise duration score − 1) × exercise frequency, yielding a possible range of 0–100, with higher scores indicating greater PA. The three components represent distinct behavioural dimensions of PA and are combined multiplicatively to derive the overall activity score, rather than functioning as interchangeable reflective indicators of a conventional psychological construct. Cronbach’s α coefficients were 0.75, 0.72, and 0.76 at T1, T2, and T3, respectively.

#### 3.3.3 Psychological flexibility

PF was assessed using the seven-item Acceptance and Action Questionnaire-II (AAQ-II) (67), translated and validated among Chinese university students by Cao et al. (68). Items are rated from 1 (“never true”) to 7 (“always true”). Because the original scoring direction represents greater experiential avoidance and lower PF, all items were reverse-scored using 8 minus the original response and summed (range 7–49); Higher scores therefore indicate lower experiential avoidance, operationalized in the present study as greater PF. Accordingly, the AAQ-II score was interpreted as reflecting lower experiential avoidance as an indicator of greater PF, rather than the full multidimensional construct of psychological flexibility. Cronbach’s α coefficients were 0.78 at T1, T2, and T3.

#### 3.3.4 Psychological resilience

PR was assessed using the 10-item Connor–Davidson Resilience Scale (CD-RISC-10) (69). The scale evaluates adaptation to change, coping with adversity, and recovery from stress. Items are rated from 0 (“not true at all”) to 4 (“true nearly all the time”) and summed (range 0–40), with higher scores indicating greater PR. Cronbach’s α coefficients were 0.81, 0.83, and 0.80 at T1, T2, and T3, respectively.

#### 3.3.5 Body dissatisfaction

BD was assessed using the 27-item short form of the Negative Physical Self Scale (NPSS-SF), adapted from the measure developed for Chinese adolescents and young adults (70). The scale comprises overall body dissatisfaction (3 items), fat dissatisfaction (9 items), appearance dissatisfaction (7 items), and shortness dissatisfaction (8 items). Items are rated from 1 (“completely inconsistent with me”) to 5 (“completely consistent with me”) and summed (range 27–135); higher scores indicate greater BD. Cronbach’s α coefficients were 0.87 at each wave.

### 3.4 Data collection

Data were collected using Wenjuanxing, a widely used Chinese online survey platform, through which electronic questionnaires, survey links, and QR codes were generated. Before formal data collection, the questionnaire battery was reviewed by the research team and pilot-tested among female university students who were not included in the final sample. The pilot test assessed item clarity, questionnaire length, technical functionality, and approximate completion time. Minor modifications were subsequently made to the instructions and questionnaire presentation without altering the content or scoring procedures of the validated measures.

After permission was obtained from the participating universities, trained research assistants introduced the study purpose, voluntary nature of participation, confidentiality procedures, and follow-up arrangements to eligible students. Survey links and QR codes were then distributed through class-based communication groups. To enable longitudinal matching while preserving anonymity, each participant created a unique identification code according to standardised instructions and used the same code at all three waves.

All questionnaire items were set as mandatory to minimise item-level missing data. Two instructed-response attention-check items were embedded in each wave to assess response attentiveness. For example, participants were instructed to select a specified response option for a designated item. Questionnaires were considered invalid if they met any of the following predefined criteria: duplicate submission; failure on either attention-check item; completion time shorter than 180 s; excessively repetitive response patterns across unrelated items; obvious logical inconsistencies in demographic information; or an identification code that could not be reliably matched across waves.

Data from each wave were screened independently before longitudinal matching. Participants who failed the predefined data-quality criteria or whose identification codes could not be reliably matched across the three waves were excluded from the final longitudinal analytic sample. Participants were informed that participation was voluntary, that they could discontinue the survey at any time without penalty, and that participation or withdrawal would not affect their academic assessment. All data were encrypted, stored on password-protected devices, and accessible only to authorised members of the research team.

### 3.5 Statistical analysis

Statistical analyses were conducted using IBM SPSS Statistics version 26.0, Mplus version 8.3, and the PROCESS macro version 3.5. Descriptive statistics, including means, standard deviations, skewness, and kurtosis, were calculated for the study variables. Internal consistency was evaluated using Cronbach’s α. Pearson correlation coefficients were used to examine the bivariate associations among T1 PA, T2 PF, T3 PR, and T3 BD. Participant attrition was evaluated by comparing baseline characteristics between participants who completed all three waves and those who did not, using independent-samples t tests for continuous variables and chi-square tests for categorical variables.

Harman’s single-factor test was conducted separately at each wave as a supplementary assessment of potential common-method variance. Longitudinal measurement invariance was evaluated across the three waves at the configural, metric, scalar, and strict levels using maximum likelihood (ML) estimation in Mplus 8.3. Model fit was assessed using the comparative fit index (CFI), Tucker–Lewis index (TLI), root-mean-square error of approximation (RMSEA), and standardised root-mean-square residual (SRMR). Measurement invariance was considered supported when the absolute change in CFI between successive models was no greater than.010. Because all questionnaire items were mandatory and the final analytic sample included only participants with complete and successfully matched data across all three waves, no item-level missing data were present in the variables included in the primary analyses. For PA, the measurement-invariance results were interpreted cautiously because the PARS-3 comprises three distinct behavioural components that are combined multiplicatively to form the overall PA score. In addition, its three-indicator configural model was just identified (df = 0), and the resulting perfect fit indices were therefore not interpreted as evidence of exceptionally strong empirical model fit.

The hypothesised indirect-association model was examined using PROCESS Model 6 with ordinary least-squares regression. T1 PA was entered as the predictor, T2 PF as the first statistical intermediary variable, T3 PR as the second statistical intermediary variable, and T3 BD as the outcome. Only-child status, BMI category, and self-rated health were included as covariates based on their theoretical and empirical relevance to PA, psychological functioning, and body-related evaluations. Only-child status was coded as 0 = no and 1 = yes; BMI category was coded as 1 = underweight, 2 = normal weight, 3 = overweight, and 4 = obesity; and self-rated health was coded as 1 = poor/fair, 2 = good, and 3 = very good. Indirect associations were evaluated using 5,000 bootstrap resamples and were considered statistically significant when their 95% bootstrap confidence intervals excluded zero. Standardised regression coefficients, standard errors, confidence intervals, and two-tailed p values were reported.

Because T3 PR and T3 BD were assessed concurrently and earlier levels of PF, PR, and BD were not controlled in the model, the estimated associations were interpreted as theoretically ordered, partially time-lagged indirect associations rather than causal mediation effects. Statistical significance was set at *p* <.05.

## 4 Results

### 4.1 Participant characteristics

The final analytic sample comprised 1,086 female university students aged 17–21 years (M = 18.32, SD = 0.71). Of these participants, 611 (56.26%) were freshmen, and 475 (43.74%) were sophomores. Regarding academic major, 28.36% studied humanities and social sciences, 21.64% natural sciences, 19.98% engineering, 13.90% medicine, and 16.11% arts or physical education. More than half of the participants were from rural areas (54.42%) and were not only children (66.39%). Most participants had a normal BMI (59.94%), perceived their family economic status as moderate (66.30%), and rated their health as good (60.50%) or very good (26.89%). Approximately one-quarter participated in university sports clubs (26.52%). Regarding monthly living expenses, 43.74% reported RMB 1,001–1,500, followed by RMB 1,501–2,000 (23.85%), RMB 1,000 or less (23.66%), and more than RMB 2,000 (8.75%). Detailed sociodemographic characteristics are presented in **Table 1**.

**Table 1:** Sociodemographic characteristics of the participants (N = 1,086).

Variable	Category	n (%)	M ± SD
Age (years)	—	—	18.32 ± 0.71
Academic year	Freshman	611 (56.26)	—
	Sophomore	475 (43.74)	—
Major	Humanities and social sciences	308 (28.36)	—
	Natural sciences	235 (21.64)	—
	Engineering	217 (19.98)	—
	Medicine	151 (13.90)	—
	Arts/physical education	175 (16.11)	—
Place of residence	Urban	495 (45.58)	—
	Rural	591 (54.42)	—
Only-child status	Yes	365 (33.61)	—
	No	721 (66.39)	—
BMI category	Underweight	219 (20.17)	—
	Normal weight	651 (59.94)	—
	Overweight	174 (16.02)	—
	Obesity	42 (3.87)	—
Perceived family economic status	Low	210 (19.34)	—
	Moderate	720 (66.30)	—
	High	156 (14.36)	—
Self-rated health	Poor/fair	137 (12.62)	—
	Good	657 (60.50)	—
	Very good	292 (26.89)	—
Participation in university sports clubs	Yes	288 (26.52)	—
	No	798 (73.48)	—
Monthly living expenses	≤ RMB 1,000	257 (23.66)	—
	RMB 1,001–1,500	475 (43.74)	—
	RMB 1,501–2,000	259 (23.85)	—
	RMB 2,000	95 (8.75)	—

BMI, body mass index; M, mean; SD, standard deviation.

### 4.2 Common method bias assessment

Given that PA, PF, PR, and BD were assessed using self-report measures, both procedural and statistical approaches were adopted to minimise and evaluate the potential influence of common method bias. At the procedural level, participation was anonymous and voluntary, and participants were informed that there were no correct or incorrect answers and that their responses would be used solely for research purposes. The confidentiality of all responses was emphasised to reduce evaluation apprehension and socially desirable responding. Standardised instructions were used across the three measurement waves, and the four scales were presented in separate sections with clear transitions. The instruments also employed different response formats and scoring procedures, including 5-point, 7-point, and 0–4 response scales, which helped reduce response-pattern consistency. Two instructed-response attention-check items were included at each wave, and questionnaires with failed attention checks, implausibly short completion times, extensive invariant responding, or obvious logical inconsistencies were excluded. In addition, the three assessments were separated by 6-month intervals, which introduced temporal separation between the variables used in the longitudinal mediation model and reduced the likelihood that the observed associations were attributable solely to same-occasion measurement.

At the statistical level, Harman’s single-factor test was conducted separately at T1, T2, and T3 following Podsakoff et al. (71). All PA, PF, PR, and BD items at each wave were entered into an unrotated exploratory factor analysis. Seven factors had eigenvalues greater than 1 at each wave. The first unrotated factor accounted for 19.13% of the total variance at T1, 20.85% at T2, and 21.10% at T3. These values were below the prespecified 40% heuristic, suggesting that no single factor dominated item covariance; However, because Harman’s single-factor test is only a preliminary diagnostic, these results should not be interpreted as ruling out the presence of common method variance.

### 4.3 Descriptive statistics and correlations

As shown in **Table 2**, the study variables exhibited acceptable distributional characteristics, with skewness and kurtosis values within acceptable ranges. T1 PA was positively correlated with T2 PF (*r* = .426, *p* <.01) and T3 PR (*r* = .419, *p* <.01) and negatively correlated with T3 BD (*r* = −.379, *p* <.01). T2 PF was positively correlated with T3 PR (*r* = .531, *p* <.01) and negatively correlated with T3 BD (*r* = −.473, *p* <.01). T3 PR was also negatively correlated with T3 BD (*r* = −.528, *p* <.01).

**Table 2:** Means, standard deviations, skewness, kurtosis, and correlations among the study variables.

Variable	M	SD	Skewness	Kurtosis	1	2	3	4
1. T1PA	27.727	28.569	1.110	0.284	1			
2. T2PF	32.923	8.109	-0.207	-0.705	.426**	1		
3.T3PR	21.698	7.464	-0.087	-0.592	.419**	.531**	1	
4.T3BD	80.086	16.130	-0.157	-0.266	-.379**	-.473**	-.528**	1

**P<0.01.

### 4.4 Longitudinal measurement invariance

As shown in **Table 3**, the configural, metric, scalar, and strict invariance models for PF, PR, and BD demonstrated acceptable-to-excellent fit across the three waves. CFI and TLI values ranged from.986 to 1.000, RMSEA values ranged from.000 to.017, and SRMR values ranged from.018 to.049. Absolute changes in CFI between successive models ranged from.000 to.004, all below the recommended.010 criterion (72, 73), supporting strict longitudinal measurement invariance for PF, PR, and BD.

**Table 3:** Three-wave longitudinal measurement invariance of the study measures.

Scale	Model	χ²	df	CFI	TLI	RMSEA	SRMR	ΔCFI
PA	Configural invariance	0	0	1	1	0	0	—
	Metric invariance	1.49	4	1	1	0	0.009	0
	Scalar invariance	4.712	8	1	1	0	0.009	0
	Strict invariance	14.245	14	1	1	0.002	0.012	0
PF	Configural invariance	82.707	42	0.991	0.986	0.017	0.049	—
	Metric invariance	76.743	54	0.995	0.994	0.011	0.043	0.004
	Scalar invariance	93.175	66	0.994	0.994	0.011	0.043	−0.001
	Strict invariance	108.127	80	0.994	0.995	0.01	0.043	0
PR	Configural invariance	101.529	105	1	1	0	0.018	—
	Metric invariance	116.345	123	1	1	0	0.024	0
	Scalar invariance	137.914	141	1	1	0	0.024	0
	Strict invariance	151.299	161	1	1	0	0.024	0
BD	Configural invariance	1036.46	954	0.995	0.994	0.005	0.025	—
	Metric invariance	1084.72	1000	0.995	0.995	0.005	0.027	0
	Scalar invariance	1139.18	1046	0.994	0.994	0.005	0.029	−0.001
	Strict invariance	1215.63	1100	0.993	0.993	0.006	0.031	−0.001

CFI, comparative fit index; TLI, Tucker–Lewis index; RMSEA, root-mean-square error of approximation; SRMR, standardised root-mean-square residual.

For PA, the metric, scalar, and strict models also showed satisfactory fit, with negligible changes in CFI across successive models. However, because the PARS-3 comprises three distinct behavioural components—intensity, duration, and frequency—that are combined multiplicatively to derive the overall PA score, its invariance results should not be interpreted as directly equivalent to those obtained for conventional multi-item reflective psychological scales. In addition, the PA configural model was just identified (*df* = 0), resulting in perfect fit indices by definition; therefore, the CFI, TLI, RMSEA, and SRMR values for this model do not provide independent evidence of exceptionally strong empirical model fit.

### 4.5 Serial mediation analysis

As shown in **Table 4**, after controlling for only-child status, BMI category, and self-rated health, the regression models comprising the hypothesised indirect-association model were statistically significant. T1 PA was positively associated with T2 PF (*β* = .419, *t* = 15.175, *p* <.001), and the model explained 18.6% of the variance in T2 PF. T1 PA (*β* = .239, *t* = 8.649, *p* <.001) and T2 PF (*β* = .434, *t* = 15.705, *p* <.001) were both positively associated with T3 PR, with the model explaining 32.9% of its variance. In the T3 BD model, T1 PA (*β* = −.127, *t* = −4.583, *p* <.001), T2 PF (*β* = −.225, *t* = −7.541, *p* <.001), and T3 PR (*β* = −.355, *t* = −11.973, *p* <.001) were all negatively associated with T3 BD, and the model explained 36.5% of its variance.

**Table 4:** Regression analysis of variable relationships in the serial mediation model.

Variables	T2 PF		T3 PR		T3 BD	
	*β*	*t*	*β*	*t*	*β*	*t*
T1 PA	0.419	15.175***	0.239	8.649***	−0.127	−4.583***
T2 PF	—	—	0.434	15.705***	−0.225	−7.541***
T3 PR	—	—	—	—	−0.355	−11.973***
*R²*	0.186		0.329		0.365	
*F*	61.849***		105.99***		103.245***	

***P<0.001.

As shown in **Table 5**, the total association between T1 PA and T3 BD was significant (β = −.371, 95% CI [−.426, −.316], *p* <.001). After T2 PF and T3 PR were included in the model, the direct association between T1 PA and T3 BD remained significant (*β* = −.127, 95% CI [−.182, −.073], *p* <.001). Based on 5,000 bootstrap resamples, the total indirect association was significant (*β* = −.243, Boot SE = .019, 95% Boot CI [−.280, −.206]) and accounted for approximately 65.5% of the total association. All three specific indirect associations were statistically significant because their 95% bootstrap confidence intervals excluded zero. The indirect association through T2 PF was *β* = −.094 (95% Boot CI [−.121, −.069]), accounting for 25.3% of the total association. The indirect association through T3 PR was *β* = −.085 (95% Boot CI [−.109, −.061]), accounting for 22.9%, while the theoretically ordered indirect association through T2 PF followed by T3 PR was *β* = −.064 (95% Boot CI [−.081, −.050]), accounting for 17.3%. These findings were consistent with significant indirect associations through PF, through PR, and through the theoretically ordered PF–PR sequence between T1 PA and T3 BD.

**Table 5:** Mediation analysis of T2 PF and T3 PR in the relationship between T1 PA and T3 BD.

Effect pathway	Effect value	Boot SE	Effect proportion	LLCI	ULCI
Direct effect: T1 PA → T3 BD	−0.127	0.028	35%	−0.182	−0.073
T1 PA → T2 PF → T3 BD	−0.094	0.013	25%	−0.121	−0.069
T1 PA → T3 PR → T3 BD	−0.085	0.012	23%	−0.109	−0.061
T1 PA → T2 PF → T3 PR → T3 BD	−0.064	0.008	17%	−0.081	−0.050
Total indirect effect	−0.243	0.019	65%	−0.280	−0.206
Total effect	−0.371	0.028	100%	−0.426	−0.316

LLCI, lower limit of the 95% confidence interval; ULCI, upper limit of the 95% confidence interval. Effect proportions were calculated by dividing the absolute value of each effect by the absolute value of the total effect. Bootstrap confidence intervals for the indirect effects were based on the PROCESS bootstrap procedure. An indirect effect was considered statistically significant when its 95% bootstrap confidence interval did not include zero.

## 5 Discussion

Using data from 1,086 female university students assessed across three waves over 12 months, this study examined the prospective association between PA and BD and the theoretically ordered indirect associations involving PF and PR. Higher T1 PA was associated with greater T2 PF and T3 PR, while T1 PA, T2 PF, and T3 PR were each negatively associated with T3 BD. Significant indirect associations were observed through PF, through PR, and through the theoretically ordered PF–PR sequence, together accounting for 65.50% of the total association. Overall, the findings suggest that adaptive psychological resources may be relevant to the prospective association between higher PA and lower later BD, while not establishing a causal mediation process.

### 5.1 PA and subsequent BD: a potentially protective but conditional association

Consistent with H1, higher T1 PA was associated with lower T3 BD. This finding is consistent with Campbell and Hausenblas’s (17) meta-analysis of exercise interventions, which reported modest improvements in body image, and with a female-specific meta-analysis showing an overall beneficial effect on body esteem (16). It also accords with broader evidence indicating that PA and sport participation are more often associated with favourable body-image outcomes (74). Because the present study assessed negative physical self rather than positive body image or body esteem, these findings should be viewed as conceptually consistent rather than directly equivalent.

One possible explanation is that PA provides experiences of movement, strength, coordination, and competence that may shift attention from appearance-based evaluation toward body functionality (75). This interpretation is consistent with objectification theory, which proposes that habitual body surveillance contributes to negative body-related evaluation (35). Through greater engagement with bodily function and competence, PA may broaden the basis of self-evaluation beyond appearance alone.

However, the association between PA and BD is likely to depend on how and why individuals engage in activity. Homan and Tylka (36) found that exercise frequency was positively related to several favourable body-image outcomes among college women, but these associations were weaker when appearance-based exercise motivation was high. Similarly, experience-sampling evidence suggests that appearance-motivated exercise may produce smaller improvements in body satisfaction and greater appearance awareness (38). The present negative association between PA and BD should therefore not be interpreted as indicating that all forms of PA are equally beneficial.

This qualification is particularly relevant because the PARS-3 assesses exercise intensity, duration, and frequency but does not capture activity type, context, motivation, or compulsivity. Students with similar PA scores may therefore have substantially different exercise experiences. The observed association should consequently be interpreted as an average relationship across potentially heterogeneous forms and motives of PA.

Reverse or reciprocal associations also remain plausible. Longitudinal evidence suggests that lower body satisfaction may sometimes precede greater activity among weight-concerned college women (19), while excessive exercise and BD may reinforce one another over time (20). Thus, the present findings support a prospective association between higher PA and lower subsequent BD, but they do not establish a unidirectional protective effect.

### 5.2 PF as a proximal self-regulatory pathway

Consistent with H2, higher T1 PA was associated with greater T2 PF. In addition, consistent with H3, a significant indirect association between T1 PA and T3 BD was observed through T2 PF, accounting for approximately 25% of the total association. This was the largest of the three specific indirect associations examined. However, because prior levels of PF and BD were not controlled, these findings should not be interpreted as showing that PA produced within-person increases in PF or that PF caused subsequent changes in BD.

One possible explanation is that PA repeatedly exposes individuals to internal experiences that cannot always be immediately controlled, including fatigue, discomfort, frustration, self-consciousness, and fluctuations in performance. Continuing to engage in meaningful activity despite such experiences may provide opportunities to practice acceptance, present-moment attention, and flexible behavioural adjustment (76, 77). In this sense, PA may provide a behavioural context associated with lower experiential avoidance and more flexible responding.

Lower experiential avoidance, operationalized in the present study as greater PF, may be particularly relevant to female university students because body-related stress often involves self-critical thoughts, appearance comparisons, and concerns about evaluation by others. More flexible responding may help students experience these thoughts without treating them as definitive judgments of personal worth or allowing them to dominate behavioural choices. This may be one reason why greater PF was associated with lower subsequent BD in the present model.

Previous evidence is broadly consistent with this interpretation. Fang et al. (24) found that cognitive defusion and PF were associated with negative body image and its improvement among Chinese university students receiving an ACT-based intervention. A meta-analytic review further showed that body-image flexibility was associated with lower body-image concerns, eating pathology, and psychological distress (45).More recent cross-lagged evidence from Chinese adults also suggested bidirectional associations between body-image flexibility and later psychological and eating-related difficulties (26). The present findings extend this literature by identifying a time-lagged indirect association involving PF between earlier PA and later BD, while the bidirectional evidence cautions against a strictly unidirectional interpretation.

Measurement considerations further qualify these findings. The AAQ-II primarily assesses experiential avoidance and psychological inflexibility rather than the full multidimensional construct of PF. Accordingly, the reverse-scored AAQ-II used in the present study should be interpreted as reflecting lower experiential avoidance, operationalized as greater PF. Future research should therefore combine the AAQ-II with multidimensional measures of PF and body-image-specific instruments such as the BI-AAQ (25).

### 5.3 PR as a broader adaptive resource

Consistent with H4, higher T1 PA was associated with greater T3 PR. Consistent with H5, a significant indirect association between T1 PA and T3 BD was observed through PR, accounting for approximately 23% of the total association. PR also showed the strongest association with T3 BD in the final regression model. However, because PR and BD were assessed concurrently at T3, these findings do not establish whether greater PR preceded lower BD or whether lower BD was associated with greater PR.

One possible explanation is that PA provides repeated opportunities to manage challenge, discomfort, setbacks, and social evaluation. Such experiences may be associated with greater mastery, confidence, and adaptive coping. Organised or group-based PA may additionally provide social support, belonging, and positive feedback, which may contribute to broader psychological resources for managing stress.

Previous longitudinal and experimental evidence is broadly consistent with this interpretation. Studies among college students have linked exercise with later PR and suggested that PR may be involved in indirect associations between PA and mental-health outcomes (28). Experimental evidence has also shown that structured PA can improve PR and that changes in PR are associated with improvements in depression and anxiety. For body image specifically, Liu et al. (29)reported a within-person indirect association involving PR between self-esteem and later body image in a three-wave random-intercept cross-lagged model. The present findings extend this literature by identifying a PA–PR–BD association, although they do not directly replicate prior work because earlier studies focused on different antecedents or outcomes.

PR should not, however, be interpreted as an individual obligation to tolerate harmful appearance pressures. Female students’ body concerns are embedded in broader social contexts, including social-media imagery, peer comparison, weight stigma, and gendered appearance standards. Consistent with the PRISM framework (56), efforts to strengthen resilience should therefore be considered alongside interpersonal support and broader environmental or systemic approaches.

The concurrent assessment of PR and BD also leaves open the possibility of reciprocal association. Lower BD may itself be related to greater PR by reducing persistent self-criticism and appearance-related stress. Future studies using additional waves and models capable of separating stable between-person differences from within-person change, such as random-intercept cross-lagged or latent-change models, are needed to clarify the temporal ordering between PR and BD.

### 5.4 From PF to PR: a sequential adaptive pathway

Consistent with H6, a significant sequential indirect association was observed from T1 PA to T3 BD through T2 PF and T3 PR, accounting for approximately 17% of the total association. Although this indirect association was smaller than those involving PF or PR alone, the pattern was consistent with the theoretical distinction between the two psychological resources. PF may reflect more immediate regulatory responding to difficult internal experiences, whereas PR may represent a broader capacity for adaptation and recovery across challenges.

From this perspective, greater PF may be associated with greater subsequent PR. Individuals who are more willing to experience discomfort, remain less entangled with negative thoughts, and continue values-consistent action may have greater opportunities to develop confidence and adaptive coping resources over time. Such repeated flexible responding may therefore be relevant to broader resilience, although the present findings do not establish that PF causes subsequent increases in PR.

Existing intervention evidence supports the modifiability of PF but does not directly establish the proposed PF-to-PR ordering. A three-level meta-analysis of ACT interventions among university students reported improvements in PF and psychological inflexibility (31), while a large web-based ACT trial also found improvements in mental health and ACT-related processes (32). However, neither study examined PR as a subsequent statistical intermediary. The significant association between T2 PF and T3 PR observed in the present study is therefore consistent with the proposed ordering, but should be regarded as theoretically informative rather than evidence of a confirmed developmental sequence.

Alternative configurations remain plausible. Greater PR may facilitate more flexible responding to discomfort, while other resources, such as self-esteem, perceived competence, social support, and emotion regulation, may contribute to both PF and PR. Lower BD may also be associated with later increases in PA or adaptive psychological resources. Future research should therefore compare the proposed PF-to-PR ordering with alternative and reciprocal models, ideally using additional waves and approaches such as random-intercept cross-lagged or latent-change models that distinguish within-person change from stable between-person differences (78, 79).

### 5.5 Practical implications

The present findings suggest that university initiatives may benefit from considering not only the amount of PA but also the context and motivation surrounding participation. Because appearance-based exercise motives have been associated with less favourable body-image outcomes, programmes could emphasise enjoyment, body functionality, competence, social connection, autonomy, and personally meaningful reasons for activity, while avoiding excessive emphasis on weight monitoring, appearance comparison, or body transformation.

PA programmes could also consider incorporating brief elements related to lower experiential avoidance and more flexible responding, such as present-moment attention during movement, distancing from body-critical thoughts, willingness to experience temporary discomfort, and clarification of personally valued reasons for activity. These components may be suitable for integration into physical-education courses, campus exercise groups, or digital wellness programmes, although their effectiveness in reducing BD should be evaluated directly in future intervention studies.

Resilience-oriented elements may provide an additional avenue for supporting students who experience appearance-related stress. Programmes could combine graded activity goals, reflection on mastery experiences, adaptive coping strategies, and peer support, while also addressing recovery from appearance criticism and social comparison. Importantly, resilience should not be framed as an individual responsibility to tolerate harmful appearance pressures; individual-level support should be accompanied by efforts to reduce weight stigma and appearance-based evaluation within the wider university environment.

Finally, support may need to be matched to students’ level of risk. Universal programmes could promote enjoyable and function-oriented PA, whereas students reporting persistent BD, rigid or compensatory exercise, weight-based self-worth, or marked avoidance may require more targeted psychological support. Screening and intervention efforts should therefore distinguish health-promoting PA from compulsive or appearance-driven exercise. Given the observational nature of the present study, these implications should be viewed as directions for programme development and future testing rather than as evidence of established intervention effects.

### 5.6 Strengths, limitations and future directions

Several strengths should be acknowledged. The study followed a relatively large sample of female university students from three universities in two Chinese provinces across three waves over 12 months. Attrition analyses revealed no significant baseline differences between retained and non-retained participants on the measured characteristics, providing no evidence of systematic attrition based on the variables examined. PF, PR, and BD demonstrated strict longitudinal measurement invariance according to established change-in-fit criteria, while the PA invariance results suggested temporal consistency but were interpreted cautiously because of the distinctive structure and scoring of the PARS-3. The study also examined two theoretically distinguishable psychological resources simultaneously and quantified their independent and theoretically ordered indirect associations.

First, temporal ordering was incomplete. PR and BD were both assessed at T3, and the reported models did not adjust for prior levels of PF, PR, or BD. Accordingly, the estimated coefficients represent theoretically ordered, partially time-lagged between-person associations rather than evidence of within-person change or causal mediation. A four-wave design separating T1 PA, T2 PF, T3 PR, and T4 BD would provide clearer temporal separation. Future studies should also consider autoregressive structural equation models, latent-change models, or random-intercept cross-lagged panel models to distinguish stable between-person differences from within-person dynamics.

Second, all focal constructs were assessed using self-report measures. Although temporal separation and procedural safeguards were adopted, Harman’s single-factor test cannot establish the absence of common method variance. Recall bias, social desirability, shared response tendencies, and general negative affectivity may therefore have influenced the observed associations. Future research should incorporate objective PA measures, such as accelerometry or wearable devices, process-oriented measures of flexibility, and ecological momentary assessment of body-image experiences.

Third, the PARS-3 captures exercise intensity, duration, and frequency but does not assess activity type, context, social climate, motivation, or compulsivity. These characteristics may meaningfully alter the association between PA and body image. Future studies should therefore assess health-, enjoyment-, functionality-, appearance-, and compensatory motives, together with excessive exercise and exercise avoidance.

Fourth, PF was operationalized as lower experiential avoidance using the reverse-scored AAQ-II. Because the AAQ-II does not capture the full multidimensional structure of PF, the present findings should not be interpreted as applying equally to all PF processes. Future studies should combine the AAQ-II with multidimensional measures of PF and body-image-specific instruments such as the BI-AAQ to determine whether domain-specific flexibility contributes explanatory value beyond lower experiential avoidance and general psychological distress.

Fifth, BD was represented by the total NPSS-SF score, which combines overall, fat, appearance, and shortness dissatisfaction. These dimensions may have different antecedents and may not be equally related to PA. For example, functional experiences associated with PA may be more relevant to weight- or function-related concerns than to height or facial appearance. Future longitudinal studies should therefore examine whether the observed associations involving PF and PR vary across specific dimensions of negative physical self.

Sixth, several potentially important confounders were not included, including appearance-focused social-media use, thin-ideal internalisation, appearance comparison, weight stigma, eating pathology, depression, anxiety, sleep, family or peer appearance commentary, and exercise motivation. The observed indirect associations may therefore partly reflect unmeasured sociocultural or psychological factors. Future research should incorporate a broader set of theoretically relevant covariates and compare alternative explanatory models.

Finally, convenience sampling from three universities in Jiangxi and Henan Provinces and the exclusive inclusion of first- and second-year female students limit generalisability. The findings may not extend to students in other regions, senior university students, non-university populations, men, or gender-diverse individuals. Replication using probability-based sampling, more geographically and culturally diverse samples, and adequately powered tests of gender and regional heterogeneity is warranted.

## 6 Conclusion

In summary, higher PA was prospectively associated with lower later BD among female university students, with significant theoretically ordered indirect associations involving PF and PR. These indirect associations accounted for approximately 65% of the total association. However, because prior levels of PF, PR, and BD were not controlled and PR and BD were assessed concurrently at T3, the findings should not be interpreted as evidence of causal mediation. The results highlight the potential value of promoting enjoyable, function-oriented PA alongside support for flexible responding and resilience, while also addressing compulsive exercise and appearance-based stigma.

## Data Availability

The original contributions presented in the study are included in the article/supplementary material. Further inquiries can be directed to the corresponding author.
